# Selection of Reference Genes for Transcriptional Analysis of Edible Tubers of Potato (*Solanum tuberosum* L.)

**DOI:** 10.1371/journal.pone.0120854

**Published:** 2015-04-01

**Authors:** Roberta Fogliatto Mariot, Luisa Abruzzi de Oliveira, Marleen M. Voorhuijzen, Martijn Staats, Ronald C. B. Hutten, Jeroen P. Van Dijk, Esther Kok, Jeverson Frazzon

**Affiliations:** 1 Food Science Department, Food Science and Technology Institute, Federal University of Rio Grande do Sul, Porto Alegre, Rio Grande do Sul, Brazil; 2 Genetics Department, Federal University of Rio Grande do Sul, Porto Alegre, Rio Grande do Sul, Brazil; 3 RIKILT, Wageningen UniversityR, Wageningen, The Netherlands; 4 Plant Breeding, Wageningen UR, Wageningen, The Netherlands; Huazhong Agricultural University, CHINA

## Abstract

Potato (*Solanum tuberosum*) yield has increased dramatically over the last 50 years and this has been achieved by a combination of improved agronomy and biotechnology efforts. Gene studies are taking place to improve new qualities and develop new cultivars. Reverse transcriptase quantitative polymerase chain reaction (RT-qPCR) is a bench-marking analytical tool for gene expression analysis, but its accuracy is highly dependent on a reliable normalization strategy of an invariant reference genes. For this reason, the goal of this work was to select and validate reference genes for transcriptional analysis of edible tubers of potato. To do so, RT-qPCR primers were designed for ten genes with relatively stable expression in potato tubers as observed in *RNA-Seq* experiments. Primers were designed across exon boundaries to avoid genomic DNA contamination. Differences were observed in the ranking of candidate genes identified by *geNorm*, *NormFinder* and *BestKeeper* algorithms. The ranks determined by *geNorm* and *NormFinder* were very similar and for all samples the most stable candidates were *C2*, exocyst complex component sec3 (*SEC3*) and *ATCUL3/ATCUL3A/CUL3/CUL3A* (*CUL3A*). According to *BestKeeper*, the importin alpha and ubiquitin-associated/ts-n genes were the most stable. Three genes were selected as reference genes for potato edible tubers in RT-qPCR studies. The first one, called *C2*, was selected in common by *NormFinder* and *geNorm*, the second one is *SEC3*, selected by *NormFinder*, and the third one is *CUL3A*, selected by *geNorm*. Appropriate reference genes identified in this work will help to improve the accuracy of gene expression quantification analyses by taking into account differences that may be observed in RNA quality or reverse transcription efficiency across the samples.

## Introduction

A wide range of biological processes leads to changes in mRNA transcription levels, and these variations are important to ensure timely cellular responses. Based on this, mRNA transcriptional profiling has become a popular research field in functional genomics studies, as it can be used to evaluate complex regulatory gene networks [1–3]. Reverse transcriptase quantitative polymerase chain reaction (RT-qPCR) has been commonly used to analyze gene expression in different organisms and under numerous conditions, since it permits specific and reproducible quantification of nucleic acids [1, 4]. However, the stability of the expressed housekeeping gene is a fundamental factor in the appropriate standard normalization of the data, which is usually normalized to more than one reference gene to avoid differences in complementary DNA (cDNA) quantity, purity, RNA stability, and enzymatic efficiency of cDNA synthesis and subsequent PCR amplifications [5–6]. The assortment of an appropriate reference gene is an absolute requirement to minimize non-biological variation between samples and achieve precise results [7]; hence, the selection of suitable reference genes is crucial to RT-qPCR analysis. The ideal reference gene would be stably expressed through all examined samples [8–9].

Many reference genes have already been identified for several crops under different treatments and conditions, particularly for model plants [10]. However, the expression of putative reference genes differs across individual sets of organs and experimental conditions [7, 11]. In this context, several free software packages such as *geNorm* [12], *NormFinder* [9] or *BestKeeper* [13], may be used in order to the best internal controls from a group of candidate normalization genes for a specific set of biological samples.

The goal of this study was to examine by RT-qPCR the stability of ten putative reference genes selected from *RNAseq* experiments. We have focused the investigation of control genes by evaluating the expression variability of 10 genes with relatively high stability levels in potato tubers.

## Materials and Methods

### Ethics statements

The field experiments in both years (in this case normal yield trials) were performed on a trial field in the proximity of Wageningen (GPS coordinates: 51.95230, 5.63490) owned by Wageningen UR. No specific permission was required to carry out these potato trials.

### Field experimental design

Eight potato edible tubers from four distinct genotypes, experimental lines, obtained in duplicates, one grown in 2011 and the other in 2012, with a post-harvest storage time of 13 and 28 days, and cultivated at Plant Breeding Sciences—Wageningen University and Research Center (WUR)—Wageningen, The Netherlands.

The varieties HZ 94 DTA 11 and RH00-386-2 are diploid, and the varieties RH4X-029-2 and RH4X-036-11 are tetraploid potato breeding clones. Although, all 4 clones have a wild potato species clone as a grandparent, they are all considered and treated as “normal” potatoes (*Solanum tuberosum*).

All potato samples are listed and detailed on Table 1.

**Table 1:** Field information of the eight potato samples used in this study for experimental validation of candidate reference genes.

**Sample ID**	**Varieties**	**Parents**	**Grand parents**	**Year of Harvest**	**Time Post-harvest (days)**
HZ-2	HZ94DTA11	RH90-012-2 x RH89-039-16	RH87-217-34 **x** TAR 24717–4 (S. tarijense)	2011	13
HZ94-2	HZ94DTA11		BC 1034 **x** SUH 2293	2012	28
RH00-2	RH00-386-2	RH97-649-11 x 96-2039-10	IVP92-057-17 **x** SPG 15458-B18 (S. spegazzinii)	2011	13
RH386-1	RH00-386-2		RH89-050-25 **x** RH89-035-38	2012	28
RH-029-2	RH4X-029-2	M 94-110-2 x FRIESLANDER	93-71-3 (S. hougasii) **x** W 72-38-720	2011	13
RH29-2	RH4X-029-2		GLORIA **x** 74 A 3	2012	28
RH036-1	RH4X-036-11	M 94-125-1 x FRESCO	BILDTSTAR **x** 93-114-5 (S. fendleri)	2011	13
RH36-1	RH4X-036-11		CEB 60-15-28 **x** PROVITA	2012	28

### Samples preparation

Four average sized tubers were selected; of these, opposite eights were pooled to minimize variation effects in the tuber. Potato tubers were washed in water at room temperature dried with paper and chopped using a food processor into 1 cm^3^ cubes. Potato cubes were immediately frozen in liquid N_2_ to avoid tuber oxidation, packed in plastic bags and stored in an ultra-freezer at -80°C. Samples were sent to ZIRBUS Technology, Tiel, The Netherlands, for lyophilisation, milling and vacuum packaging. Potato powder was stored at room temperature until use.

### RNA isolation and quality assessment

RNA was isolated from 0.5 g of each freeze-dried sample, according to the hexadecyltrimethylammonium bromide (CTAB) buffer lysis method, followed by chloroform/isoamyl alcohol extraction and overnight precipitation with lithium chloride (LiCl) proposed by van Dijk et al. (2009) [14], with some modifications, as follows. Lysis was performed with the extraction buffer pre-warmed to 60°C before use; the chloroform/isoamyl alcohol extraction was repeated three times before the LiCl precipitation; and the final precipitation with 96% ethanol was performed with the tubes kept on ice and then centrifuged at 4°C for 15 min at 14,000 g. Total RNA isolated was dissolved in 100 μL of 10 mM Tris (pH 7,0) and warmed to 65°C for 10 min. Total RNA was stored at -80°C until use.

RNA purity and concentration were assessed by absorbance measurements using a Nanodrop 1000 instrument (Thermo Fisher Scientific, NanoDrop Technologies Wilmington, DE, USA). For integrity evaluation, 1 μg of RNA was migrated by electrophoresis (10 min at 80 V and 50 min at 100 V) in denaturing agarose gel (1% agarose, 5% formamide, 1X TBE) stained with ethidium bromide. Gels were visualized in Gel Doc XR+ Systems (Bio-Rad Laboratories, Life Technologies Corporation, Carlsbad, CA, USA) and analyzed using Quantity One 1-D (Bio-Rad Laboratories).

### Candidate gene selection and primer design

Candidate potato reference genes with stable expression levels in tubers were selected from a large collection of *RNAseq* profiles generated for 90 potato tubers grown under diverse range of growth conditions, locations, and growth year. Ten potato genes with more than 50 counts per million reads and with lowest interquartile range (IQR) were selected using R version 3.01 [15] for further evaluation with RT-qPCR (Table 2). Information about candidate genes was determined using Ensembl Plant Database (http://plants.ensembl.org/index.html).

**Table 2:** Candidate potato reference genes with more than 50 counts per million reads (highest expression), lowest inter quartile ranges (IQRs) and known functions, used for experimental validation.

**Gene**	**IQR**	**Gene Code**	**Location**	**Transcript Code**	**Forward/Reverse primer**	**Amplicon (bp)**	**PCR efficiency (%)**	**r** ^2^
eukaryotic translation initiation factor 3 subunit	12.84	PGSC0003DMG400009231	11:9004475–9012212	PGSC0003DMT400023872	3’GCGAAGATCCCAGTGAACAA5’	123	93.6	0.998
					5’CAGCATCTTCACCAGCACTTA3’			
dead-box atp-dependent rna helicase 39*	17.25	PGSC0003DMG400023195	12:54853693–54861561	PGSC0003DMT400059671	3’TATGGGTGCCAAAGGGAAAG5’	116	86	0.998
				PGSC0003DMT400059672	5’CGTCTACTGAGAGAGACTCCAA3’			
3-oxoacyl-(acyl-carrier protein) reductase	17.61	PGSC0003DMG401026981	6:52692660–52698742	PGSC0003DMT400069374	3’AGTTGAAGCTCCGGTTGTTATT5’	100	96.9	0.998
					5’GTTCACAAGGACCTTACAACCA3’			
importin subunit alpha	17.66	PGSC0003DMG400007289	6:100326–106781	PGSC0003DMT400018802	3’ACCTCGATAAGAAGCTGGAGA5’	100	96	0.996
				PGSC0003DMT400018803	5’AGTTTCCGGAACTGTGTTGT3’			
exocyst complex component sec3	17.75	PGSC0003DMG402015451	12:56757079–56759688	PGSC0003DMT400039945	3’GGAGCAGTATATCCAAGGACAA5’	75	90.3	0.995
					5’AGGAACATTGTAGTGACAAACTTAG3’			
*ATCUL3/ATCUL3A/CUL3/CUL3A*	17.80	PGSC0003DMG400001321	2:46264503–46268790	PGSC0003DMT400003337	3’GAGGACCGGTGAAGTGATAAAC5’	120	90	0.994
				PGSC0003DMT400003338	5’TCAGCCGAGACATCAAGAAAC3’			
				PGSC0003DMT400003339				
ubiquitin-associated/ts-nTS-N domain-containing protein	19.03	PGSC0003DMG402005949	6:54204271–54209252	PGSC0003DMT400015247	3’TGAGAAGGCTGAAGAGACTTTG5’	131	101	0.996
				PGSC0003DMT400015248	5’GTAAGTTCTGGGTGGTGGTATT3’			
*C2*	19.90	PGSC0003DMG400023712	10:57539858–57542161	PGSC0003DMT400060959	3’GGCCACTCAGATTGTCTCTATG5’	118	88,1	0.998
					5’AGCTTTGCTTCTCCTCATACTC3’			
dck/dgk-like deoxyribonucleoside kinase	20.49	PGSC0003DMG400009278	11:13898057–13903160	PGSC0003DMT400023985	3’GATATTGAAGCAAAGAGGCAGTATG5’	112	95.5	0.997
					5’GATTGCCCTTAGGCTGTTCT3’			
2-isopropylmalate synthase b	21.25	PGSC0003DMG400016337	6:39132151–39142170	PGSC0003DMT400042133	3’AAAGTGGCATCCATCAGGA5’	104	104.5	0.998
					5’GACAATACCAGATTTATTAGCACGA3’			

*Gene with alternative-splicing isoforms.

*Solanum tuberosum* genes, cDNA sequences, and exon-intron-exon junctions were also obtained from Ensembl Plant Database. All primers were designed using the Primer Quest tool from IDT DNA (http://www.idtdna.com/primerquest/Home/Index) with melting temperatures between 58°C and 62°C, GC contents from 45 to 65% and amplicon lengths ranging from 75 to 150 bp (Table 2). The Oligo Analyzer software from IDT DNA was also used to infer primer secondary structures (http://www.idtdna.com/analyzer/applications/oligoanalyzer/).

Since 4 out of the 10 candidate genes display alternative splicing (see Table 2), BLAST searches were performed, in order to design oligonucleotides complementary to a region of homology between the different transcripts of a given gene.

To determine PCR efficiencies, standard curves were constructed with four points in five-fold dilutions starting from a 1/5 cDNA concentration (1:5, 1:25, 1:125 and 1:625), according to Perini, et al. (2014) [16] and strongly suggested by Bustin, et al. (2009) [17]. Reaction efficiencies (E) and correlation coefficients (r^2^) were estimated using *StepOne Software v*.*2*.*3* (*Life Technologies*), based on the slopes of the plots and the Cps (crossing points) versus log input of cDNA. E and r^2^ values for each reaction performed are also presented in Table 2.

### Complementary DNA synthesis

Each RNA sample was converted into cDNA in triplicates, as recommended by Bustin, et al. (2009) [17]. One microgram of total RNA was used for synthesis according to the manufacturer´s protocol, using the *iScript* cDNA Synthesis Kit (BIORAD). Specificity of the primers was checked for the 24 resulting cDNAs by end-point PCR followed by electrophoresis in agarose gel and melting curve analysis. The cDNA samples were stored at -20°C until use.

### Quantitative PCR (qPCR)

qPCR chain reactions were carried out in a *StepOne* Plus Real Time PCR System (Life Technologies) using SYBR Green (BIORAD; 1:10,000 dilution) for monitoring double strand DNA synthesis during qPCR. Reactions were performed in a 20 μL final volume with 10 μL of diluted cDNA (1:50), 0.2 μM of each primer, 0.1 mM of dNTPs, 0.25 units of Platinum Taq DNA Polymerase (Life Technologies) 1X Buffer Solution, and 1.5 mM of MgCl_2_. Each cDNA was analyzed in four technical replicates, and negative controls were included. PCR cycling conditions were as follows: 94°C for 5 min, 40 cycles at 94°C for 15 seconds, 60°C for 10 seconds, 72°C for 15 seconds and 60°C for 35 seconds, and a final melting curve between 50 and 99°C (Δ0.3°C/s).

### Gene expression stability analyses

All results from RT-qPCR were compared using *NormFinder* [9], *geNorm*—v. 3.5 [12] software and *BestKeeper* an Excel-based program [13].

The *NormFinder* algorithm ranks candidate genes based on their stability of expression and determines the best pair of genes for using as endogenous controls for the samples. *geNorm* calculates the average expression stability (M-value), defining the mean variation of a certain gene in relation to the other candidate genes. Following, *geNorm* determines the best number of reference genes through the pairwise variation estimation (V). Vandesompele et al. (2002) [12] suggested a V cut-off value of 0.15, below which the inclusion of an additional reference gene would not be required. Finally, *BestKeeper* estimates the reference genes with the greatest expression stability by assessing a *BestKeeper* Index specific for each sample, which is calculated as the geometric mean of the Cp values of its candidate housekeeping genes [13].

## Results

### RT-qPCR analysis of candidate reference genes

In order to select a reliable set of reference genes for gene expression studies in potato edible tubers, RT-qPCR assays were performed for 10 candidate housekeeping genes. The correlation coefficients (r^2^) for all resulting amplification curves were higher than 0.99, and all 10 primer pairs allowed amplification efficiencies (E) between 86 and 104.5% (Table 2). Considering the optimal PCR efficiency as 100%, which allows duplication of the whole target cDNA at each PCR cycle during the exponential phase, the observed efficiency values were considered acceptable; hence, the amplification products of each reaction were comparable to each other.

Primers for elongation factor 1 alpha, *18S rRNA* [18, 19], and actin [20] genes were initially included in the data set; however, they were discarded from the analysis due to unexpected amplification products.

**Fig 1:**
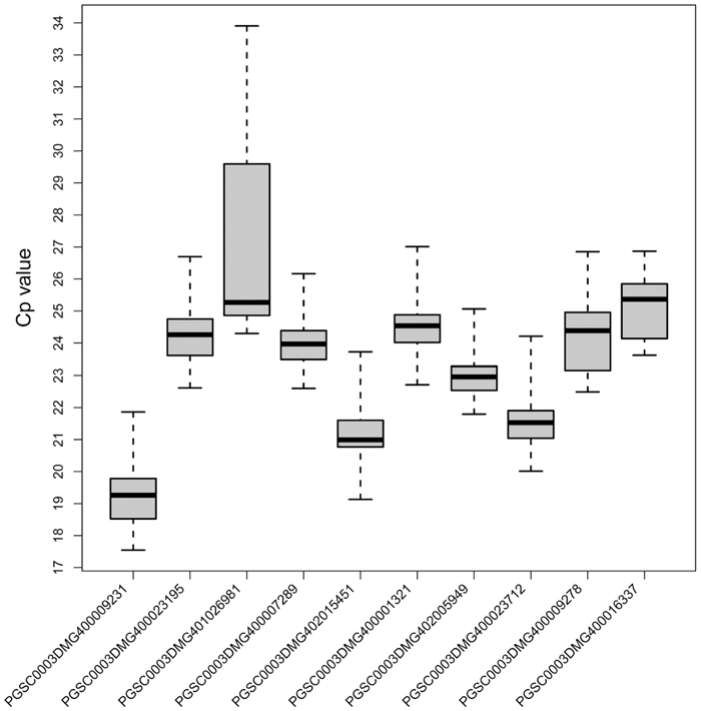
Expression profiles of the tested reference genes in raw Cp values for all 8 samples. Expression data are displayed as raw Cp values for each reference gene across all samples. The line denotes the median and boxes indicate the 25/75 percentiles.

Next, Cp values [21] were used to analyze the steady state mRNA levels of each candidate gene in eight different potato samples, showing a relative wide range of Cp values (Fig 1). In all tested samples, the lowest mean Cp value was observed for the gene eukaryotic translation initiation factor 3 subunit, followed by exocyst complex component sec3 (*SEC3*).

### Analyses of reference genes stability via *geNorm*, *NormFinder* and *BestKeeper* algorithms

Three different algorithms, *NormFinder*, *geNorm* and *BestKeeper*, were applied in computational assessment of gene expression stability in order to minimize potential biases intrinsic to each software.

*NormFinder* uses a mathematical modelling that allows an estimation of gene expression based in a variation of reference genes and in a subgroup of sample sets, considering the best genes those with the lowest stability value, with minimal intra and inter group variation, and indicate the best combination of gene pairs groups and subgroups [9]. Table 3 presents the ranking of the candidate reference genes according to their stability value for the samples, as determined by *NormFinder*. This algorithm identified *C2*, followed by *SEC3*, as the most stably expressed genes in all 8 different samples.

**Table 3:** Ranking of candidate reference genes according to the estimated values of stability of expression, as calculated by the *NormFinder* algorithm and M value calculated using *geNorm* estimated M–values, for the candidate reference genes.

**Gene**	**Gene Code**	**Rank by *NormFinder***	**Stability by *NormFinder***	**M-value by *geNorm***
*C2*	PGSC0003DMG400023712	1	0,010	0.647
exocyst complex component sec3	PGSC0003DMG402015451	2	0,015	0.716
*ATCUL3/ATCUL3A/CUL3/CUL3A*	PGSC0003DMG400001321	3	0,016	0.658
dead-box atp-dependent rna helicase 39	PGSC0003DMG400023195	4	0,018	0.736
ubiquitin-associated /ts-n domain-containing protein	PGSC0003DMG402005949	5	0,019	0.756
importin subunit alpha	PGSC0003DMG400007289	6	0,019	0.709
dck/dgk-like deoxyribonucleoside kinase	PGSC0003DMG400009278	7	0,021	0.829
2-isopropylmalate synthase b	PGSC0003DMG400016337	8	0,021	0.763
eukaryotic translation initiation factor 3 subunit	PGSC0003DMG400009231	9	0,024	0.734
3-oxoacyl-(acyl-carrier protein) reductase	PGSC0003DMG401026981	10	0,084	3.222
-	-	Best combination of 2 genes (*SEC3* and *C2*)	0,010	**-**

*Lowest M value by *geNorm*.

**Fig 2:**
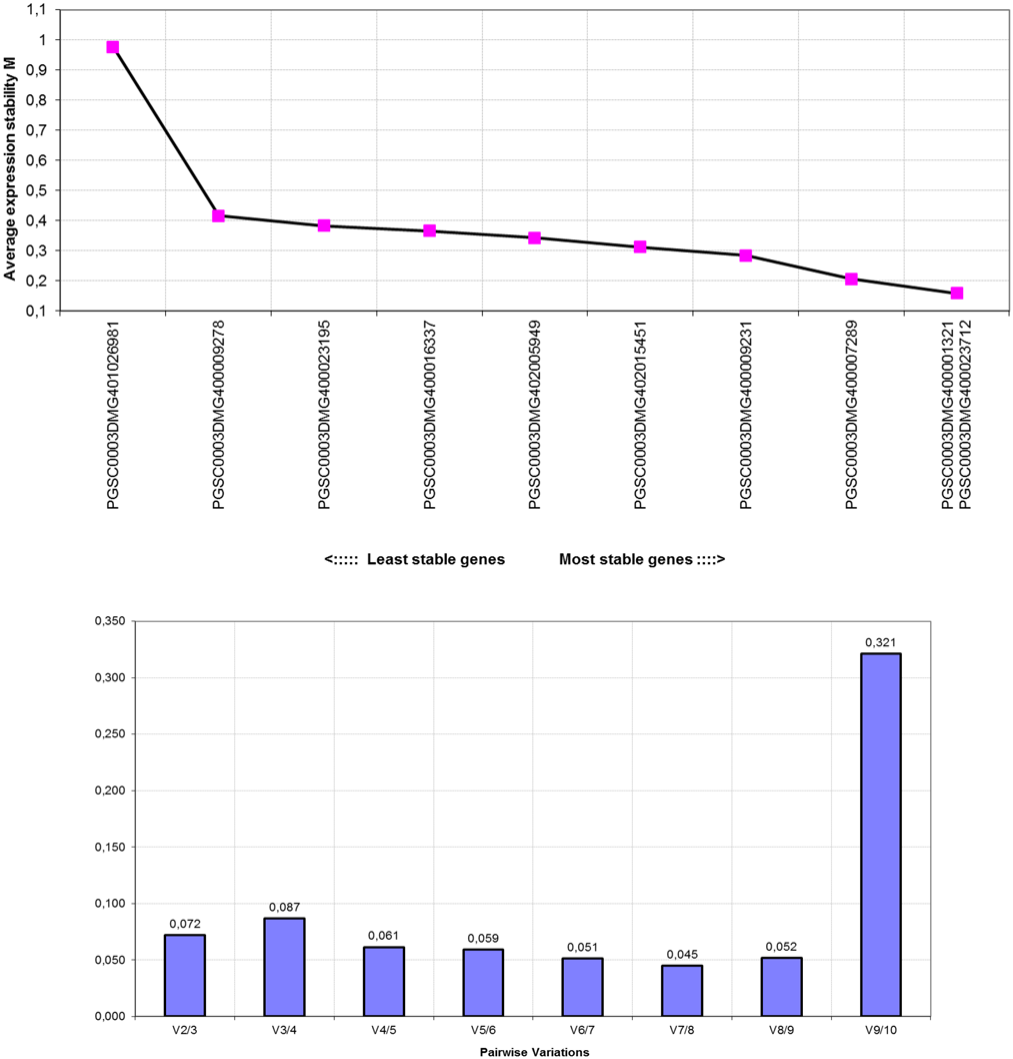
Average values of stability of gene expression for the selected reference genes assessed by *geNorm*. The plots indicate expression profiles and the determination of the optimal number of control genes for the eight samples.

Table 3 describes the ranking of candidate genes as assessed by *geNorm*. Also, pairwise variations (V) were calculated for obtaining the optimal number of normalization factors and the use of 2 primer pairs were definitively enough for this dataset. Fig 2 shows the M-values and pairwise variation (V) calculated by *geNorm* for all candidates and their best partners for the potato samples. The most stable candidate gene was *C2*, followed by *ATCUL3/ATCUL3A/CUL3/CUL3A* (*CUL3A*), with M-values above 0.7 for both. In agreement, the best gene pair consisted also of the *C2* and *CUL3A* (see Fig 2). Additionally, the V-values were below the established 0.15 threshold suggested by Vandesompele et al. (2002) [12], corroborating that the inclusion of an additional gene is not required for data normalization.

According to the *BestKeeper* algorithm, the importin subunit alpha and ubiquitin-associated/ts-n domain-containing protein genes were the most stably expressed ones in *S*. *tuberosum* edible tubers across all eight samples, with a standard deviation (SD) of 0.66 for both candidates (Table 4). Only 3-oxoacyl-(acyl-carrier protein) reductase and dck/dgk-like deoxyribonucleoside kinase (less stable) were considered to be inconsistent for *BestKeeper* quality parameters ([±Cp] > 1.00), with SDs 1.01 and 3.05, respectively.

**Table 4:** Descriptive statistics of candidate reference gene expression patterns, as measured by BestKeeper.

**Gene**	**Gene Code**	**Geometric Mean [CP]**	**Arithmetic Mean [CP]**	**min [CP]**	**max [CP]**	**Standard Deviation [± CP]** ^a^	**Coefficient of Variation [% CP]**	**min [x-fold]**	**max [x-fold]**	**Standard Deviation [± x-fold]** ^a^
eukaryotic translation initiation factor 3 subunit	PGSC0003DMG400009231	19.33	19.37	17.55	21.86	0.87	4.48	-3.45	5.77	1.83
dead-box ATP-dependent RNA helicase 39	PGSC0003DMG400023195	24.14	24.17	22.61	26.70	0.85	3.52	-2.89	5.87	1.80
3-oxoacyl-(acyl-carrier protein) reductase	PGSC0003DMG401026981	26.93	27.14	24.30	33.90	3.05^a^	11.24	-6.19	126.03	8.29
importin subunit alpha	PGSC0003DMG400007289	24.08	24.10	22.60	26.16	0.66b	2.72	-2.80	4.24	1.58
exocyst complex component sec3	PGSC0003DMG402015451	21.17	21.20	19.13	23.73	0.82	3.87	-4.12	5.86	1.77
*ATCUL3/ATCUL3A/CUL3/CUL3A*	PGSC0003DMG400001321	24.60	24.62	22.71	27.01	0.71	2.88	-3.71	5.32	1.63
ubiquitin-associated /ts-n domain-containing protein	PGSC0003DMG402005949	23.04	23.06	21.79	25.06	0.66^b^	2.87	-2.38	4.06	1.58
*C2*	PGSC0003DMG400023712	21.65	21.67	20.01	24.21	0.72	3.34	-3.11	5.91	1.65
dCK/dgk-like deoxyribonucleoside kinase	PGSC0003DMG400009278	24.31	24.34	22.49	26.85	1.01^a^	4.13	-3.54	5.83	2.01
2-isopropylmalate synthase b	PGSC0003DMG400016337	25.16	25.17	23.62	26.87	0.81	3.20	-2.90	3.28	1.75

^a^Genes with standard deviations [±Cp] > 1.00 are considered to have inconsistent expression patterns (3-oxoacyl-(Acyl-carrier protein) reductase and dck/dgk-like deoxyribonucleoside kinase).

^b^Based on the standard deviations (SDs), genes can be ranked from most stably (lowest SD, importin subunit alpha and ubiquitin-associated /ts-n domain-containing protein) to least stably (highest SD, 3-oxoacyl-(acyl-carrier protein) reductase) expressed.

## Discussion

Recently, the quantification of RNA transcripts has become increasingly rapid and precise due to advances in gene quantification strategies. Associated with that, newly identified reference genes showing more stable expression patterns than traditional normalization genes have been reported by analyzing microarray and transcriptome sequencing data [22–23], and these high throughput techniques might be excellent potential sources of good candidate reference genes, as showed in the present work.

The accuracy of RT-qPCR results is highly dependent on a reliable normalization strategy that employs an invariant (i.e. stably expressed) reference gene [24–25]. For example, Nicot et al. (2005) and Lopez-Pardo, Ruiz de Galarreta and Ritter (2013) [18, 19] already performed this analysis testing several reference genes, including the elongation factor 1 alpha, with successfully results. Different of our data, the analysis was based on candidates chosen from the literature, not on gene expression experiments, such as microarray or *RNAseq*. In addition, Nicot et al. (2005) [18] did not use samples derived from edible tubers, but samples from a pool of all parts of the potato plant, both under biotic and abiotic stresses, without any distinction between different plant organs. Still, Lopez-Pardo, Ruiz de Galarreta and Ritter (2013) [19] used potato edible tubers as samples, but specifically under cold stress.

It has become clear that no single gene is constitutively expressed in all cell types and under all experimental conditions. For instance, the expression of the so-called ‘housekeeping’ genes, although constant under some experimental conditions, can vary considerably in other cases, implying that the stability of the proposed control gene has to be tested before each new experiment [7, 10, 11, 16, 26–28]. Normalization with multiple reference genes is becoming a common practice and the gold standard for the technique, but reports that identify such genes in plant investigations are still limited [7, 16, 18, 28–41].

In the present work we evaluated by RT-qPCR 10 reference genes displaying relatively stable expression in edible tubers. Results obtained by *geNorm* and *NormFinder* were very similar to each other and more different than those obtained by *BestKeeper*. While the *geNorm* and *NormFinder* algorithms correct for inter-sample variations, *BestKeeper* does not regard differences in RNA quality or cDNA conversion efficiency across samples, which might influence the distinct findings observed here. Differently from the pairwise approach used by *geNorm*, *NormFinder* selects the top rank candidates with minimal variation rather than correlated expression, which is less influenced by co-regulated genes. Moreover, *NormFinder* takes into consideration systematic differences between sample subgroups [9, 12–13]. Hence, it is expected that the comparison of these three algorithms, as performed here, might provide a more reliable set of reference genes under specific experimental conditions. In this sense, our study provides evidence for the use of certain genes as normalizers in gene expression experiments for potato edible tubers, which is essential for obtaining accurate and reliable gene expression data profiles.

From our analysis three genes called *SEC3*, *CUL3A* and *C2* were selected as the best normalizers in gene expression of potato edible tubers. However, for each set of samples a validation are needed, and the best reference gene may be different, this could be observed in this present work that the rank for ten candidates to be reference genes for our 8 samples were not exactly the same order of the rank as RNAseq database.

The gene *SEC3* as well as *SEC5*, *SEC6*, *SEC8*, *SEC10*, *SEC15*, *EXO70*, and *EXO84* genes are part of an evolutionarily conserved octameric protein complex of secretory vesicles [42–43]. The *Arabidopsis* genome encodes single or multiple isoforms of all exocyst subunits [44], and homologous structural models of plant exocyst subunits indicate well conserved rod-like structural features, including putative phosphatidylinositol phosphate binding sites on SEC3 and EXO70 subunits. Through interaction with RAB and RHO GTPases, these proteins are known to be crucial for the proper targeting of the exocyst to membranes [45].

The *CUL3* gene is a constituent of ubiquitin ligase complexes [40]. In *Arabidopsis*, both CUL3A and CUL3B proteins interact with the RING-H2 finger protein RBX1 and with several members of plant BTB domain proteins [46–47], suggesting that they form similar CUL3-based E3 complexes. However, *cul3a* loss-of-function mutants are viable and fertile, exhibiting only slightly delayed flowering and reduced sensitivity to far red light [46]. This viability might be attributed to functional redundancy between the two *CUL3* genes in *Arabidopsis*, since disruption of both genes causes embryo lethality, indicating that CUL3 plays important roles during early steps of plant development [48–49]. Indeed, CUL3 seems to regulate the ethylene-independent distal root patterning and primary root growth by a novel ethylene-dependent pathway, thus implicating CUL3 in the division and organization of the root stem cell niche and columella root cap cells [50].

Finally, the gene that is referred to in the EnsemblPlants database and hence in this paper as *C2* is actually coding for a yet uncharacterized protein, designated M1C6S3_SOLTU in the UniProt database (http://www.uniprot.org/uniprot/M1C6S3). In this entry it is mentioned that the protein contains three C2 domains. The C2 domain polypeptide is one of the most prevalent eukaryotic lipid-binding domains used in diverse functional contexts. This structural domain helps target proteins to cell membranes, and its typical version (PKC-C2) has a beta-sandwich conformation composed of 8 β-strands that co-ordinate two or three calcium ions, which bind in a cavity formed by the first and final loops of the domain on the membrane binding face [50–51].

## Conclusions

Transcriptome data such as those obtained from microarray and *RNAseq* experiments provide an excellent resource of selecting candidate RT-qPCR reference genes. Here, through bioinformatics and experimental data, we show the selection and validation of ten putative reference genes for RT-qPCR studies in potato samples. The *C2*, *SEC3*, and *CUL3A* genes were found to be the most stable and suitable normalizers for potato edible tubers expression studies. In summary, these findings provide useful tools for the normalization of RT-qPCR experiments and will enable more accurate and reliable gene expression studies related to functional genomics in potato.
